# Unraveling the Immune Landscape of Chronic Obstructive Pulmonary Disease: Insights into Inflammatory Cell Subtypes, Pathogenesis, and Treatment Strategies

**DOI:** 10.3390/ijms26073365

**Published:** 2025-04-03

**Authors:** Chou-Chin Lan, Mei-Chen Yang, Wen-Lin Su, Kuo-Liang Huang, Ching-Chi Lin, Yi-Chih Huang, Chun-Yao Huang, Hsin-Yi Chen, Chih-Wei Wu, Chung Lee, Lun-Yu Jao, Yao-Kuang Wu

**Affiliations:** 1 Division of Pulmonary Medicine, Taipei Tzu Chi Hospital, Buddhist Tzu Chi Medical Foundation, New Taipei City 231, Taiwanwilliamsu2007@gmail.com (W.-L.S.);; 2 School of Medicine, Tzu-Chi University, Hualien 970, Taiwan

**Keywords:** chronic obstructive pulmonary disease, inflammatory cells, pathogenesis

## Abstract

Chronic obstructive pulmonary disease (COPD) is a prevalent respiratory disorder characterized by persistent airway inflammation and progressive airflow limitation, resulting in a significant global health burden and high mortality. This narrative review synthesizes the current evidence on the roles of leukocyte subtypes—including neutrophils, monocytes, lymphocytes, eosinophils, and basophils—in the pathogenesis and clinical management of COPD. Relevant original studies and reviews are included, providing data on leukocyte functions, associated biomarkers, and therapeutic implications. Neutrophils contribute to airway damage and remodeling by releasing proteases and reactive oxygen species, particularly in response to environmental exposure such as cigarette smoke or air pollution. Lymphocytes, especially CD8⁺ T cells, drive chronic inflammation and immune dysregulation. Monocytes differentiate into macrophages that promote airway fibrosis and persistent inflammation, further impairing lung function. Eosinophils, though classically linked to asthma, are now recognized for their role in eosinophilic COPD, where they are associated with an increased exacerbation risk and corticosteroid responsiveness. Basophils, though less studied, may influence airway inflammation through interactions with eosinophils and cytokine release. Understanding these immune cell dynamics provides insights into the heterogeneity of COPD and highlights potential targets for precision therapy. Tailored interventions based on inflammatory phenotypes may improve clinical outcomes and advance personalized treatment strategies.

## 1. Introduction

Chronic obstructive pulmonary disease (COPD) is a common respiratory disease and ranks among the leading causes of death worldwide [1]. It is characterized by chronic inflammation of the airways and lungs, and it is often triggered by smoking or inhaled irritants, leading to long-term airflow obstruction and lung damage [1]. Many individuals with COPD experience reduced exercise tolerance and a decline in their health-related quality of life [1]. Although there have been advancements in COPD treatments, patients frequently continue to struggle with symptoms such as dyspnea, cough, and sputum, which significantly affect their quality of life, even when receiving standard care [1].

The pathogenesis of COPD involves several complex mechanisms, beginning with an inflammatory response triggered by inhaled irritants, such as cigarette smoke, and pathogens [1]. Damaged epithelial cells release thymic stromal lymphopoietin (TSLP) and pro-inflammatory cytokines, such as interleukin (IL)-33, IL-25, reactive oxygen species (ROS), and C-X-C motif chemokine ligand 8 (CXCL8) [2]. This response further activates inflammatory pathways in the lungs, leading to the accumulation of inflammatory cells, which release various cytokines and mediators that perpetuate inflammation and recruit additional immune cells [2]. Chronic inflammation results in structural changes in the airways, known as airway remodeling, which is characterized by the thickening of airway walls, fibrosis, and increased mucus production because of goblet cell hyperplasia [2]. This excessive mucus secretion contributes to airway obstruction, whereas smooth muscle hypertrophy further narrows the airways, restricting airflow [2]. In emphysema, the destruction of alveolar walls occurs because of an imbalance between proteases (e.g., neutrophil elastase) and antiproteases (e.g., alpha-1 antitrypsin), leading to a reduced surface area for gas exchange, resulting in dyspnea [2]. Additionally, increased oxidative stress in the lungs exacerbates inflammation and tissue damage, perpetuating the injury cycle [2]. Overall, the multifactorial nature of COPD, involving environmental exposure and inflammatory processes, contributes significantly to its development and progression [2].

The white blood cell (WBC) differential count quantifies the relative proportions of different types of leukocytes [3]. The main types of WBCs included in the differential count are neutrophils, lymphocytes, monocytes, eosinophils, and basophils [3]. Different WBCs perform different functions. Neutrophils, the most abundant type of WBCs, work as the first defense against bacterial infections by quickly responding to sites of infection or inflammation [3]. Lymphocytes, which include T and B cells, are essential for adaptive immunity. T cells help regulate immune responses, while B cells produce antibodies that neutralize pathogens [3]. Monocytes migrate to the lungs, where they differentiate into macrophages, subsequently phagocytosing pathogens and debris. Dendritic cells present antigens to T cells, bridging the adaptive and innate immune responses [3]. Eosinophils play an important role in allergic reactions and release cytotoxic granules that can damage parasites [3]. Basophils are crucial in allergic reactions, as they release histamine and other mediators that promote inflammation and recruit other immune cells to the sites of allergic responses [3]. In the context of COPD, different WBCs have specific implications that may offer useful insights into disease mechanisms, progression, and treatment [3].

Understanding the immunological heterogeneity in COPD may provide a framework for developing phenotype-driven treatment strategies [3]. This working hypothesis is supported by accumulating evidence highlighting the association between immune cell profiles and COPD severity, exacerbation risk, and treatment response [3]. Therefore, the aim of this review is to comprehensively examine the functional roles of leukocyte subtypes in COPD, their clinical significance, and their potential as therapeutic targets.

## 2. Methods

This study presents a narrative review aiming to synthesize the current evidence on the roles of leukocyte subtypes in the pathogenesis, progression, and clinical management of COPD. We followed the general principles of a narrative review methodology and structured our search to ensure comprehensive coverage of the relevant literature. A systematic search of the literature was conducted using five electronic databases: PubMed, Scopus, Web of Science, Cochrane Library, and Google Scholar. The search period included all articles published up to January 2024. The search terms included combinations of keywords and MeSH terms, such as “COPD”, “chronic obstructive pulmonary disease”, “neutrophils”, “eosinophils”, “lymphocytes”, “monocytes”, “basophils”, “leukocytes”, “inflammation”, “biomarkers”, “phenotype” and “treatment”. Boolean operators (AND, OR) were used to refine the search.

**Figure 1:**
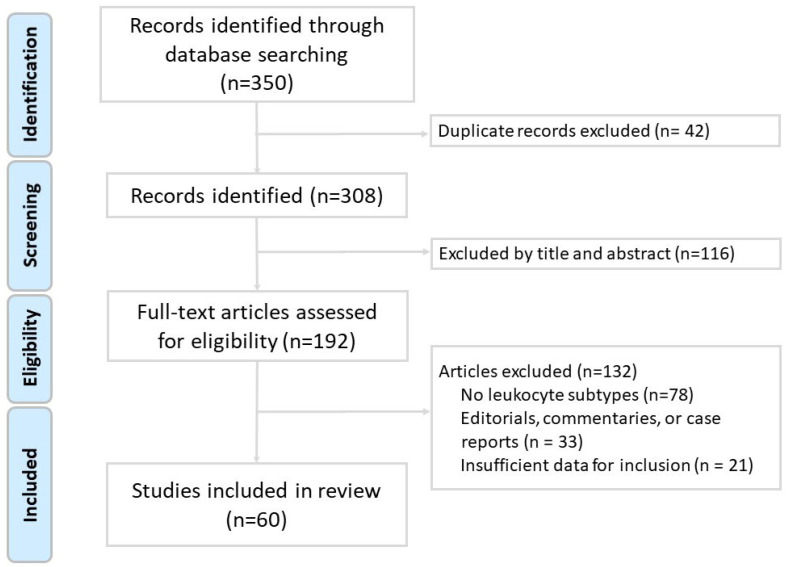
Flow diagram of literature search and article selection for this narrative review.

The inclusion criteria encompassed original research articles involving human, animal, or in vitro studies; reviews and meta-analyses relevant to the immune pathogenesis of chronic obstructive pulmonary disease (COPD); studies published in English; and articles that specifically addressed the role of leukocyte subtypes in COPD pathogenesis, inflammation, exacerbations, or therapeutic strategies. The exclusion criteria included case reports, editorials, conference abstracts, letters, and studies that were unrelated to leukocyte subtypes or not specific to COPD. Two independent reviewers screened the titles and abstracts for relevance and performed full-text evaluations of potentially eligible articles. Any discrepancies were resolved through discussion or consultation with a third reviewer. As shown in the study flow diagram (Figure 1), after screening 350 records, a total of 60 studies met the inclusion criteria and were included in the final review. We did not perform a formal risk-of-bias assessment or grade the level of evidence, as is typical for narrative reviews. However, preference was given to recent high-impact publications, systematic reviews, and clinically relevant studies. The data extracted included leukocyte functions, biomarkers, disease associations, and implications for patient management and treatment.

## 3. Results

### 3.1. The Role of Neutrophils in COPD

#### 3.1.1. Neutrophilic Inflammation in COPD Pathogenesis

Neutrophilic inflammation is a key feature of COPD, contributing to its pathogenesis, progression, and exacerbation [4]. Neutrophils are essential components of the inflammatory response to lung stimuli [4]. In COPD, neutrophils are recruited to the lungs and airways due to chronic exposure to irritants, such as cigarette smoke and environmental pollutants [4]. During this process, chemotactic factors released by macrophages contribute to increased neutrophil migration into the lungs. CXC chemokines (such as CXCL1, CXCL5, and CXCL8) and cytokines (including IL-1β and TNF-α) serve as key attractants for neutrophils [4]. These cytokines and chemokines bind to C-X-C chemokine receptors (CXCR1 and CXCR2) on the surface of neutrophils, facilitating their recruitment [4]. Once activated, neutrophils release various pro-inflammatory cytokines, chemokines, and proteases, further promoting an inflammatory environment in the lungs and recruiting additional immune cells [5]. However, this inflammatory response can lead to tissue damage, as neutrophils release proteolytic enzymes, such as neutrophil elastase and myeloperoxidase, which degrade extracellular matrix components, resulting in emphysema and airway remodeling [5]. Additionally, neutrophils generate ROS during activation, contributing to oxidative stress, which exacerbates inflammation and damages the lung tissue [5]. The excessive presence and activation of neutrophils in COPD can also impair the clearance of pathogens and debris from the airways, increasing the risk of respiratory infections and exacerbations [4]. Recent studies have suggested a role for neutrophil extracellular traps (NETs) in COPD, reporting that these may contribute to lung tissue damage and inflammation [6]. NETs also release ROS and proteases, which are cytotoxic to cells [6]. Overall, neutrophils and NETs induce inflammatory processes in COPD, influencing both the pathogenesis and progression of the disease.

#### 3.1.2. Clinical Studies of Neutrophils in COPD

A previous study defined a sputum neutrophil percentage ≥ 61% as the cutoff for neutrophilic inflammation [7]. Clinical studies have indicated that individuals who have COPD and smoke exhibit a higher number of neutrophils in their airways than non-smokers [8]. Patients with a higher sputum neutrophil percentage (>86.2%) exhibited a higher GOLD stage, increased cough severity and CAT scores, poorer lung function, greater air trapping, and more severe exacerbation [8]. Another study defined neutrophilic inflammation using a sputum neutrophil percentage cutoff of >64%, with 75% of participants with COPD exceeding this threshold [9]. The mean sputum neutrophil percentages reported were as follows: group A, 66.5% (range: 15–91); group B, 84.2% (58–99); group C, 72.1% (18–88); and group D, 78.7% (44–97). These findings indicated that patients in GOLD groups B and D tended to exhibit more pronounced neutrophilic inflammation, supporting an association between greater disease severity and elevated neutrophil levels [9]. Neutrophil polarization refers to the process by which neutrophils undergo asymmetric reorganization of their internal structure and surface receptors in response to external stimuli [10]. In patients with COPD, peripheral blood neutrophils exhibit significantly increased polarization compared with healthy controls (7.99% ± 3.91%), particularly in those with GOLD stage II (27.50%), III (24.21%), and IV (18.13%) disease [10]. Neutrophils are important in acute exacerbation, and their numbers and activities can increase substantially in such cases. One study found that 61.2% of patients with COPD experienced neutrophilic exacerbations (defined as >7000 cells/μL or >73%), 17.7% experienced eosinophilic exacerbations (defined as >200 cells/μL or >2%), and 21.1% experienced mixed-type exacerbations [11]. Mortality rates were higher in the neutrophilic exacerbation group [11].

#### 3.1.3. Potential Therapeutic Targets for Neutrophilic Inflammation in COPD

Understanding the specific role of neutrophils in COPD could lead to the development of new therapeutic strategies. As corticosteroids are less effective against neutrophil-driven inflammation, there is a need for therapies that specifically target neutrophils. Several agents have been investigated for their ability to reduce neutrophil-driven inflammation in patients with COPD. AZD8999 was found to demonstrate anti-inflammatory effects in neutrophilic COPD, achieving approximately 50% maximal inhibition of pro-inflammatory cytokines in LPS-stimulated neutrophils from patients with COPD [12]. When combined with fluticasone propionate (0.1 nM), even a low concentration of AZD8999 (0.01 nM) produced synergistic anti-inflammatory effects. These findings support the potential of AZD8999 and fluticasone as combination therapy for enhanced inflammation control in COPD [12]. In a phase 1 trial, ladarixin, another antagonist of both CXCR1 and CXCR2, was found to decrease neutrophilic inflammation during corticosteroid-resistant exacerbations caused by influenza A infection [13]. One study found that neutrophils from patients with COPD exhibited enhanced chemotaxis toward CXCL1. A phosphodiesterase-4 inhibitor, roflumilast, inhibited this chemotaxis in a concentration-dependent manner [14]. In a randomized trial, patients with COPD who received roflumilast for 12 months showed significantly greater improvements in lung function parameters and quality of life than the placebo group [15]. A 52-week, double-blind, placebo-controlled trial evaluated the efficacy of roflumilast in patients with severe-to-very severe COPD, all of whom received inhaled corticosteroid/long-acting β_2_-agonist therapy [16]. Roflumilast significantly improved lung function and reduced exacerbation rates in participants with more than three exacerbations in the previous year [16]. A 6-month trial assessed the CXCR2 antagonist MK-7123 in moderate-to-severe COPD patients and found that it significantly improved FEV_1_ (mean difference: 67 mL) and reduced sputum neutrophil counts [17].

### 3.2. The Role of Lymphocytes in COPD

#### 3.2.1. Lymphocytic Inflammation in COPD Pathogenesis

Lymphocytes also play a crucial role in the pathogenesis of COPD through their involvement in immune response and chronic inflammation. The primary lymphocyte subtypes implicated are T and B cells. Increased activated T cells, particularly CD8^+^ cytotoxic T lymphocytes, are found in the lungs and airways of patients with COPD [18]. These T cells contribute to the inflammatory response through the release of pro-inflammatory cytokines, such as interferon-gamma (IFN-γ) and tumor necrosis factor-alpha (TNF-α), which perpetuate inflammation and promote tissue damage [18]. CD4^+^ T helper cells have been shown to be involved in the immune response in COPD, further influencing the inflammatory environment [19]. Two CD4^+^ T helper (Th) cell phenotypes are involved in lung inflammation [19]: Th1 cells mainly produce IL-2, IFNγ, and TNF, while Th2 cells primarily secrete IL-4, IL-5, and IL-13. Both phenotypes play distinct roles in inflammatory responses in the lungs [19]. Th17 cells are a subset of pro-inflammatory T cells that produce IL-17, which contributes to chronic inflammation, particularly by promoting neutrophilic infiltration in the lungs [19]. B cells also play a role in COPD, particularly in the production of autoantibodies and the formation of bronchial lymphoid follicles [20]. These follicles in the lungs are associated with local inflammation and contribute to disease progression [20]. Overall, the dysregulation of lymphocyte function in COPD can lead to an imbalance between pro-inflammatory and anti-inflammatory responses, exacerbating the chronic inflammation and tissue damage that are characteristic of the disease.

#### 3.2.2. Clinical Studies of Lymphocytes in COPD

Several clinical studies have investigated the role of lymphocytes in COPD, providing insights into their involvement in disease progression.

One study using endobronchial biopsies found that CD8^+^ T-cell infiltration was significantly increased in the small airways of both smokers and patients with COPD [21]. A meta-analysis showed an increased presence of CD8^+^ T cells in lung tissues, accompanied by increased expression levels of associated cytotoxic proteins in patients with COPD [18]. COPD lungs have also been reported to exhibit increased levels of B cells and plasma cells compared with control lungs, and numbers of infiltrating B cells in the airways correlate with disease severity [20]. A peripheral blood analysis of patients with varying CAT scores [22] found that those with CAT scores > 30 exhibited markedly reduced CD4^+^ cells (21.8%) and elevated CD8^+^ cells (53.1%), resulting in the lowest CD4^+^/CD8^+^ ratio (0.43) [22]. As CAT scores decreased, CD4^+^ cell percentages increased, while CD8^+^ percentages decreased. Patients with CAT scores ≤ 10 had the highest CD4^+^ levels (41.3%), lower CD8^+^ levels (21.4%), and the highest CD4^+^/CD8^+^ ratio (2.19) [22]. These findings suggest a correlation between greater symptom burden and increased predominance of CD8^+^ T cells in peripheral blood [22].

IL-17, a pro-inflammatory cytokine secreted by Th17 cells, is elevated in both the serum and sputum of patients with COPD [23]. A meta-analysis reported significantly higher serum IL-17 levels in stable COPD patients than in controls (standardized mean difference [SMD]: 1.77) and even higher levels in AECOPD than in stable COPD (SMD: 1.78) [23]. Sputum IL-17 concentrations were also significantly increased in COPD patients relative to the controls (SMD: 2.03) [23]. These findings support IL-17 as a potential biomarker for lymphocytic inflammation and disease exacerbation in COPD [23].

Acanfora et al. used a cutoff value of 20% and found that a relatively low lymphocyte count ≤ 20% was an independent risk factor for three-year mortality in elderly patients with severe COPD [24]. In another study, lymphopenia was defined as an absolute lymphocyte count below 0.8 × 10^9^/L [25]. Patients with lymphopenia experienced higher in-hospital mortality, prolonged hospital stays, and longer durations of mechanical ventilation [25].

A high peripheral blood neutrophil-to-lymphocyte ratio (NLR), resulting from increased inflammation (higher neutrophil levels) or reduced immune function (lower lymphocyte levels), is associated with poor prognosis. Peripheral blood NLRs are elevated in patients with more severe COPD [26]. Patients with FEV_1_ < 50% were found to have a median NLR of 2.52, whereas those with FEV_1_ ≥ 50% were found to have a median NLR of 2.06. Similarly, patients with mMRC ≥ 2 had a higher NLR (2.59) than those with mMRC < 2 (2.06) [26]. A recent study found that patients who died from all causes had an NLR of 3.0 and that survivors had an NLR of 2.3, with a higher NLR significantly associated with increased all-cause mortality (hazard ratio = 1.16) [27]. These results highlight the NLR as a useful biomarker for assessing COPD severity [26]. Taylan et al. found that the mean NLR was 1.7 in healthy controls, 3.1 in stable COPD, and 7.1 in COPD exacerbation [28]. Using an NLR cutoff of 3.29, the sensitivity and specificity for detecting COPD exacerbation were 80.8% and 77.7%, respectively [28]. They suggested that an elevated NLR may indicate heightened inflammation during COPD exacerbation and could aid in the early detection of exacerbations, even in individuals with normal levels of conventional inflammatory markers [28]. The NLR has also been suggested as a simple marker for predicting the severity of exacerbations and the need for hospitalization and mechanical ventilation [29]. During exacerbations, the mean NLR was found to be 12.2, decreasing to 3.5 one month later. An NLR cutoff value of 12.585 predicted hospitalization with a sensitivity of 89.5% and a specificity of 85.7%. Additionally, a cutoff of 14.89 demonstrated 100% sensitivity and 96.2% specificity for predicting the need for mechanical ventilation [29].

#### 3.2.3. Potential Therapeutic Targets for Lymphocytic Inflammation in COPD

Ongoing clinical studies are exploring potential therapeutic targets and lymphocyte biomarkers for the management of COPD. TNF-α is a key factor in the pathogenesis of COPD, and its levels are elevated in patients with the disease [30]. Infliximab, an immunoglobulin that targets and blocks TNF-α activity, has been explored for its therapeutic potential in COPD. However, a multicenter, randomized controlled trial involving patients with moderate-to-severe COPD showed that infliximab did not significantly improve lung function or quality of life, nor did it reduce exacerbation rates compared with a placebo, despite the proposed role of TNF-α in COPD pathogenesis [31]. In COPD patients with type 2 inflammation marked by high eosinophil counts, dupilumab, an IL-4 and IL-13 blocker, was linked to fewer exacerbations and better lung function [32]. A phase 3 randomized trial evaluated dupilumab in patients with COPD and type 2 inflammation, defined by blood eosinophil counts ≥ 300 cells/μL [32]. Over 52 weeks, treatment with dupilumab (300 mg every two weeks) significantly reduced the rate of exacerbations and improved lung function compared to placebo [32]. A phase I randomized-controlled trial evaluated tozorakimab, an anti–IL-33 monoclonal antibody, in patients with mild COPD [33]. The treatment was well tolerated and significantly reduced serum IL-5 and IL-13 levels, supporting its proposed mechanism of action. However, its clinical efficacy requires further investigation in larger trials [33].

### 3.3. The Role of Monocytes in COPD

#### 3.3.1. Monocytic Inflammation in COPD Pathogenesis

Monocytes also play an important role in the pathogenesis of COPD through their involvement in inflammatory and immune responses [34]. Upon activation by various inflammatory stimuli, including cigarette smoke and environmental pollutants, monocytes migrate to the lungs and differentiate into macrophages, which are key players in the pulmonary inflammatory response [34]. Activated circulating and resident macrophages contribute to the chronic inflammation observed in the lung tissues and airways and release pro-inflammatory cytokines, such as IL-6, TNF-α, and IL-8, which recruit more immune cells, such as neutrophils or lymphocytes, to the site of inflammation [34]. Additionally, macrophages secrete matrix metalloproteinases (MMPs), which degrade the extracellular matrix, thereby contributing to tissue destruction, airway remodeling, and alveolar damage typical of emphysema [34]. Their production of ROS further exacerbates inflammation and oxidative stress. This overall inflammatory response can lead to tissue damage, impaired lung function, and disease progression. Furthermore, monocytes are involved in tissue remodeling, during which they contribute to fibrosis and structural changes in the airways, exacerbating the decline in lung function [34].

#### 3.3.2. Clinical Studies of Monocytes in COPD

A few clinical studies have investigated the phenotype and role of monocytes in COPD, thereby providing insights into their contribution to the disease. CD14^+^ monocytes derived from the peripheral blood of individuals with COPD showed an elevated production of IL-6 and CCL2 [35]. Elevated serum IL-6 levels derived from CD14^+^ monocytes were linked to an increased exacerbation frequency and inversely related to the forced expiratory volume in one second–forced vital capacity ratio [35]. A previous study involving 444 patients revealed an association between monocyte levels and COPD exacerbations. The lowest risk of exacerbation occurred when monocyte proportions ranged from 7.4% to 10%. In contrast, monocyte proportions < 7.4% with an absolute count < 0.62 × 10^9^/L or proportions > 10% were associated with significantly increased risk of exacerbation [36]. Arginase activity, a marker of monocyte activation, was elevated during COPD exacerbations (0.70 µmol urea/min) compared with normal controls (0.50 µmol urea/min), and this elevation persisted for up to three months after the exacerbation [37]. Additionally, a recent study found that both the NLR and monocyte-to-lymphocyte ratio (MLR) can serve as predictors of COPD exacerbation [38]. The MLR was found to be 0.23 in controls and 0.67 in patients with stable COPD, and it increased to 0.91 during exacerbation. Higher NLR and MLR values were associated with increased risk of exacerbation [38].

The exact mechanism linking monocyte counts to COPD exacerbations remains unclear. Although studies have shown elevated monocyte/macrophage levels in patients with COPD, these cells exhibited impaired phagocytic functions. The proportion of alveolar macrophages displaying phagocytic activity was found to be significantly lower in patients (11.6 ± 4.1%) than in controls (25.6 ± 9.2%). Such dysfunction contributes to persistent bacterial colonization, the accumulation of necrotic debris, and sustained airway inflammation [39].

#### 3.3.3. Potential Therapeutic Targets for Monocytic Inflammation in COPD

Given their involvement in inflammation and remodeling, monocytes could be potential targets for therapy for COPD. Strategies to modulate monocyte activation or inhibit inflammatory mediators may offer novel treatment options. Clinical studies targeting monocytes in COPD are limited, although some in vitro studies have explored novel treatments targeting them. Thioredoxin was found to significantly inhibit the lipopolysaccharide (LPS)-induced production of pro-inflammatory cytokines in human macrophages [40]. Additionally, elevated CCL2 expression levels were found in the lungs of mice with COPD, and these levels correlated with macrophage activation [41]. The application of a CCR2 inhibitor was found to protect against injury caused by cigarette smoke, LPS-induced lung injury, and airway remodeling [41]. Therefore, targeting the CCL2-CCR2 signaling pathway related to COPD is recommended [41]. However, as clinical studies targeting monocyte-related pathways are still limited, their clinical efficacy in COPD remains to be fully established.

### 3.4. The Role of Eosinophils in COPD

#### 3.4.1. Eosinophilic Inflammation in COPD Pathogenesis

Although neutrophilic inflammation is often prominent in COPD, eosinophilic inflammation can also occur, and this is known as eosinophilic COPD [42]. Eosinophilic COPD has a distinct phenotype characterized by the active involvement of eosinophils. Recent research has highlighted the significance of circulating eosinophils as emerging biomarkers of COPD [42].

Eosinophilic inflammation can contribute to the development and exacerbation of COPD, leading to airway remodeling, a decline in lung function, airway hypersensitivity, and more severe exacerbation [42]. Eosinophils release inflammatory mediators, such as major basic proteins, eosinophil cationic proteins, and eosinophil-derived neurotoxins [42]. These substances drive inflammation, airway remodeling, and hyperresponsiveness. Eosinophils contribute to excessive mucus production and airway obstruction [42]. The molecular basis of eosinophilic inflammation in COPD involves key cytokines, such as IL-4 and -5, which are essential for eosinophil survival and activation, and IL-13, which promotes airway hyperreactivity and mucus production [42]. Eotaxin-1 recruits eosinophils to the airways by interacting with the CCR3 receptor. IL-33 and thymic stromal lymphopoietin (TSLP) enhance the Th2 cell response and intensify eosinophilic activity [42]. Once recruited and activated, eosinophils release cytokines, such as TGF-β, which stimulate fibroblast growth, leading to fibrosis and increased collagen deposition in the airway walls [42]. Furthermore, eosinophilic substances promote the proliferation of smooth muscle cells in the airway walls, thereby thickening the airway walls, enhancing bronchoconstriction, trapping air in the lungs, and exacerbating the respiratory symptoms [42]. Furthermore, eosinophils release cytotoxic granules containing major basic proteins and eosinophil peroxidase, which can cause epithelial damage and contribute to airway hyperresponsiveness and obstruction. Eosinophils also produce cytokines involved in the pathogenesis of emphysema, thereby influencing extracellular matrix production and fibrosis, which contributes to structural changes in the lungs [42]. Additionally, eosinophils generate ROS, increase oxidative stress, further damage the lung tissue, and accelerate the progression of COPD [42]. Eosinophil-derived cytokines (such as IL-13) and chemokines attract other inflammatory cells, such as macrophages and neutrophils, and the enzymes and reactive species produced by these cells exacerbate emphysema symptoms and drive disease progression [42].

#### 3.4.2. Clinical Studies of Eosinophils in COPD

An eosinophil percentage threshold of 3% is often used to identify eosinophilic inflammation, which is identified in 22–46% of patients with stable COPD [7,43]. The GOLD classification is partially associated with distinct COPD phenotypes, with group C (now categorized as group E) showing increased eosinophilic inflammation, evidenced by a mean eosinophil count of 300 cells/µL—significantly higher than in groups A (60 cells/µL) and B (70 cells/µL) [9]. In a longitudinal study of stable COPD, higher levels of circulating eosinophils (≥170 cells/μL) predicted a greater decline in FEV1 [44]. The use of inhaled corticosteroids (ICSs) was found to be associated with slower progression of airflow obstruction [44]. Another study identified blood eosinophil counts ≥ 300 cells/µL as an independent risk factor for accelerated lung function decline [45]. Additionally, elevated blood eosinophils (≥2%) are associated with risk of exacerbations, contributing to disease progression, lung function decline, and increased disease severity [46].

Many clinical studies have demonstrated an association between blood eosinophil counts and COPD exacerbations [47]. A meta-analysis involving 79,868 participants evaluated various thresholds of high blood eosinophil counts, including absolute counts (200, 300, and 400 cells/μL) and percentages (2%, 3%, and 4%) [47]. The analysis found that elevated eosinophil levels were significantly associated with increased risk of COPD exacerbation at thresholds of 300 cells/μL (relative risk [RR]: 1.21), 400 cells/μL (RR: 1.79), 2% (RR: 1.26), and 4% (RR: 1.44). Additionally, high eosinophil counts were linked to moderate-to-severe exacerbations at thresholds of 300 cells/μL (RR: 1.30) and 2% (RR: 1.33) [47]. A meta-analysis of COPD data revealed that patients with eosinophil counts >2% had higher hospital readmission rates [48]. This highlights the complex role of eosinophils in determining disease severity and COPD outcomes.

#### 3.4.3. Potential Therapeutic Targets for Eosinophilic Inflammation in COPD

Steroids are effective in treating eosinophilic COPD, and clinical studies have suggested that eosinophil counts can guide the use of inhaled corticosteroids to reduce exacerbation rates, with one study reporting a 17% reduction in moderate-to-severe exacerbations in patients with ≥2% blood eosinophils who received inhaled corticosteroids [49]. Hospitalized patients with COPD and eosinophilic exacerbations tend to have shorter hospital stays after systemic corticosteroid treatment [50]. Therefore, the eosinophil count is a valuable parameter for predicting the effectiveness of steroids. A Cochrane review assessed the efficacy of mepolizumab and benralizumab in COPD with eosinophilia and eosinophilic inflammation [51]. Mepolizumab is a humanized monoclonal antibody that binds to IL-5, thereby preventing IL-5–mediated activation of eosinophils. In contrast, benralizumab targets the IL-5 receptor alpha (IL-5Rα) expressed on eosinophils, triggering antibody-dependent cell-mediated cytotoxicity and resulting in rapid depletion of these cells [51]. Mepolizumab significantly reduced moderate-to-severe exacerbations by 19% in patients with eosinophil counts ≥ 150/μL (RR 0.81) [51]. It also extended the time to first exacerbation. Benralizumab significantly reduced severe exacerbations requiring hospitalization in patients with eosinophils ≥ 220/μL. Improvements in quality of life were minimal and fell below clinically meaningful thresholds. Both therapies were generally safe, with no significant differences from placebo in terms of adverse events [51]. A phase 3 trial in patients with COPD and type 2 inflammation (eosinophils ≥ 300/μL) demonstrated that dupilumab (anti–IL-4/IL-13) significantly reduced the annualized rate of moderate or severe exacerbations and led to greater improvements in lung function (FEV_1_: +160 mL vs. +77 mL with placebo) and quality of life (SGRQ: −9.7 vs. −6.4). Adverse event rates were comparable between the dupilumab and placebo groups [52]. A phase 2 trial assessing the use of itepekimab, an anti-IL-33 monoclonal antibody, in patients with COPD did not meet its primary endpoint in the overall population [53]. However, a subgroup analysis revealed that, in former smokers, itepekimab significantly reduced exacerbation rates (RR 0.58) and improved lung function. No benefits were observed in current smokers. Adverse events were similar between the itepekimab and placebo groups [53].

### 3.5. The Role of Basophils in COPD

#### 3.5.1. Basophilic Inflammation in COPD Pathogenesis

Basophils are often considered to be involved in allergic responses [54]. As they occur in lower numbers than other immune cells, basophils have been largely neglected in previous studies. However, recent studies have increasingly recognized their role in the pathogenesis of COPD. Most basophils express CCR3, which is activated by RANTES (CCL5), eotaxin (CCL11), and monocyte chemotactic protein-3 (MCP-3) [55]. Shibata et al. showed that basophils played a role in the development of emphysema in an elastase-induced mouse model [54]. Basophil-derived IL-4 enhances the macrophage production of matrix metalloproteinase-12 (MMP-12), accelerating emphysema progression [54]. These macrophages contribute to the destruction of alveolar walls and lung tissue, driving the development of emphysema [54]. Additionally, basophils regulate eosinophil infiltration by producing IL-4 and IL-13, and they secrete IL-8 and CCL5, which facilitate the recruitment of macrophages, neutrophils, and eosinophils, important immune cells in the pathogenesis of COPD [54].

#### 3.5.2. Clinical Studies of Basophils in COPD

Clinical studies of basophils in COPD have been few. However, recent clinical studies have investigated the role of basophils in COPD and revealed their potential significance in the disease process. Basophils were found in lungs affected by COPD, and their counts were significantly increased in patients with severe COPD [56]. In biopsy cohorts, basophil levels were reported to be significantly elevated in GOLD stage I–III patients compared with never-smokers [56]. Additionally, basophils were more prevalent in the bronchioles of patients with GOLD stage IV disease, and their tissue density increased with disease severity [56]. A previous study revealed that basophils were dysregulated in COPD, with several basophil-related genes exhibiting increased expression levels in affected patients [57]. Furthermore, the expression of basophil-related genes was linked to eosinophilic inflammation in both the airways and blood and to poorer lung function in patients with COPD [57]. This suggests a potential role of basophils in the development of COPD. However, the role of basophils in influencing the clinical outcomes of COPD is not fully understood and remains an area of ongoing research.

#### 3.5.3. Potential Therapeutic Targets in Basophilic Inflammation in COPD

Basophils also express IL-5Rα and are impacted by the blockade of IL-5/IL-5Rα [58]. Benralizumab has been linked to significant reductions in the expression of genes associated with eosinophils and basophils, as well as genes involved in immune signaling complexes [58]. Ongoing clinical studies are essential for investigating the implications of basophilic inflammation and its potential as a therapeutic target in the management of COPD.

## 4. Overview of Leukocyte Subtypes in COPD Pathogenesis

**Figure 2:**
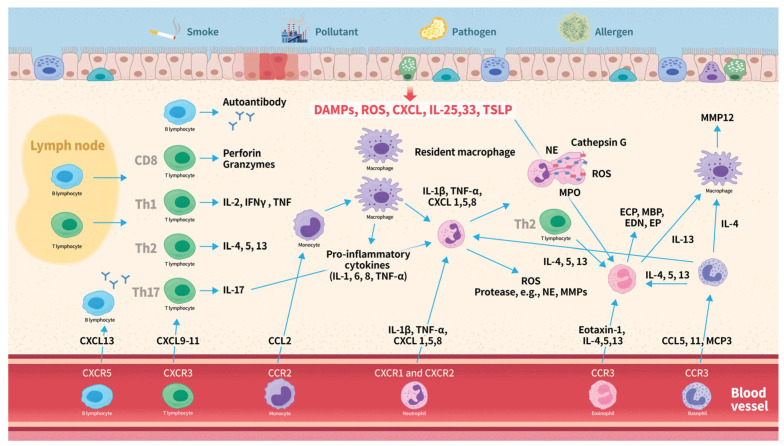
Overview of leukocyte subtypes in COPD pathogenesis. Abbreviations: CCL, C-C motif chemokine ligand; CCR, C-C chemokine receptor; CXCL, C-X-C motif chemokine ligand; CXCR, C-X-C chemokine receptor; COPD, chronic obstructive pulmonary disease; EDN, eosinophil-derived neurotoxin; ECP, eosinophil cationic protein; EP, eosinophil peroxidase; IFN-γ, interferon-gamma; IL, interleukin; MBP, major basic protein; MCP, monocyte chemotactic protein; ROS, reactive oxygen species; TSLP, thymic stromal lymphopoietin; Th, T helper cell; TNF-α, tumor necrosis factor-alpha.

The pathogenesis of COPD in relation to leukocyte subtypes is summarized in Figure 2. It often begins with inflammatory responses triggered by inhaled irritants, such as smoke, pollutants, or pathogens. Damaged epithelial cells release ROS, TSLP, IL-33 and -25, and CXCL8. This response activates inflammatory pathways, leading to inflammation and tissue damage. C-X-C chemokines serve as attractants for neutrophils and bind to CXC chemokine receptors on the surface of these neutrophils, thereby facilitating their recruitment. Activated neutrophils release ROS, cytokines, chemokines, and proteases, which further amplify the inflammatory response. Neutrophil extracellular traps contribute to lung tissue damage and inflammation. Chemotactic cytokines such as CXCL9, CXCL10, and CXCL11, recruit T lymphocytes expressing CXCR3, whereas CXCL13 recruits B lymphocytes expressing CXCR5. CD8^+^ cytotoxic T lymphocytes contribute to the inflammatory response by releasing pro-inflammatory cytokines, including IFN-γ and TNF-α, perpetuating inflammation and tissue damage. Th1 cells mainly produce IL-2, IFNγ, and TNF, while Th2 cells primarily secrete IL-4, IL-5, and IL-13. Both phenotypes play distinct roles in the inflammatory responses in the lungs. B cells produce autoantibodies that may contribute to tissue inflammation. Upon activation by various inflammatory stimuli, monocytes migrate to the lungs, where they differentiate into macrophages. Activated circulating and resident macrophages release cytokines and chemokines, which recruit additional immune cells. Eotaxin-1 recruits eosinophils to the airways by binding to the CCR3 receptor. IL-4, 5, 13, and 33, and TSLP further enhance eosinophilic activity. Eosinophils release a variety of inflammatory mediators, including IL-4, IL-5, IL-13, TGF-β, ROS, ECP, MBP, EP, and EDN. These mediators contribute to inflammation, airway remodeling, and hyperresponsiveness. Basophils express the CCR3 receptor, which is activated by CCL5, CCL11, and MCP-3. Upon activation, basophils release elastase, IL-4, IL-13, IL-8, and CCL5, which promote the recruitment of neutrophils, eosinophils, and macrophages. The activation of these different types of leukocytes triggers an inflammatory response, resulting in airway inflammation, remodeling, and tissue destruction.

## 5. Clinical Implications

Understanding the distinct roles of leukocyte subtypes in COPD pathogenesis offers valuable insights that have the potential to enhance patient management through more personalized treatment strategies. The heterogeneous inflammatory profiles observed in COPD, characterized by neutrophilic, eosinophilic, lymphocytic, monocytic, and basophilic responses, highlight the importance of immunophenotyping in guiding therapy selection and disease monitoring. The clinical significance of each leukocyte subtype in COPD is summarized in Table 1.

To facilitate clinical application, our review organizes these findings according to the specific leukocyte subtype involved and the corresponding therapeutic and prognostic implications. Neutrophil-dominant COPD, which is often associated with severe airflow limitation and corticosteroid resistance, may benefit from targeted agents such as CXCR2 antagonists (e.g., navarixin and ladarixin) or PDE4 inhibitors (e.g., roflumilast), which have shown promise in reducing neutrophilic inflammation and exacerbation [12,13,14,17]. In contrast, eosinophilic COPD represents a distinct phenotype that responds favorably to inhaled corticosteroids and biologic therapies targeting interleukin pathways—specifically IL-5 and IL-4/IL-13—such as mepolizumab, benralizumab, and dupilumab [49,50,51,52]. Lymphocytic inflammation, particularly involving CD8^+^ T cells and Th17 cells, contributes to chronic immune dysregulation and tissue damage. Biomarkers such as the NLR and MLR are increasingly recognized as useful tools for assessing systemic inflammation, predicting exacerbations, and estimating mortality risk [25,27,28,29,38]. These ratios are easily obtainable from routine blood tests, offering clinicians a practical, non-invasive means of risk stratification. Monocytes, through their differentiation into pro-inflammatory macrophages, play a crucial role in airway remodeling and persistent inflammation. Elevated CCL2 levels and CCR2 signaling have been implicated in this process, suggesting that targeting the CCL2–CCR2 axis may offer a novel therapeutic approach to attenuate monocyte-driven inflammation in COPD [41]. Furthermore, emerging evidence points to the role of basophils in COPD, especially in the more advanced stages of the disease. Basophils not only contribute to eosinophilic inflammation but also release mediators that recruit other immune cells and promote tissue remodeling. This interplay with eosinophils suggests potential for dual-targeted interventions, particularly in patients with overlapping or refractory inflammatory phenotypes [54,56,57,58].

For the clinical application of leukocyte differentiation in COPD management, consideration of the timing of WBC differential testing is essential. Complete blood count (CBC) with differential is a readily available, low-cost tool that can be utilized both during stable disease and acute exacerbations of COPD [59,60]. In stable patients, initial and periodic monitoring of leukocyte profiles can help identify the dominant inflammatory phenotype, which may guide individualized long-term treatment decisions, including the use of inhaled corticosteroids or biologic therapies [59]. During acute exacerbations, serial WBC measurements can reflect dynamic inflammatory changes, assist in distinguishing between neutrophilic and eosinophilic exacerbation phenotypes, and inform therapeutic choices [60]. Incorporating WBC differential testing into routine COPD care—particularly at the initial assessment, during follow-up visits, and in cases of acute exacerbation—may assist in key clinical decisions such as initiation or adjustment of therapy [59,60].

Taken together, these findings support a paradigm shift toward precision medicine in COPD, where treatments are selected based on the dominant immune profile rather than a one-size-fits-all approach. Integrating leukocyte subtype analysis into routine clinical practice may improve treatment efficacy, minimize unnecessary exposure to ineffective therapies, and ultimately enhance patient outcomes. Future research should prioritize the development of standardized inflammatory phenotyping tools and the identification of reliable biomarkers to guide clinical decision making.

## 6. Limitations of This Review

While this review provides a comprehensive overview of leukocyte subtypes and their roles in COPD pathogenesis and management, several limitations should be acknowledged. First, much of the current understanding is derived from observational and experimental studies, which may not fully capture the complexity of immune responses in diverse patient populations. Second, although certain biomarkers and therapeutic targets show promise, their clinical applicability may be limited by variability in measurement methods, cutoff values, and patient heterogeneity. Third, emerging evidence on basophils and other less-studied immune cells remains preliminary, requiring further validation in larger, well-controlled clinical studies. Finally, the dynamic and overlapping nature of immune responses in COPD poses challenges in defining distinct phenotypes, which may complicate the implementation of precision medicine strategies.

Although this review has certain limitations, it nonetheless provides valuable and comprehensive insights into the roles of leukocyte subtypes in COPD. By summarizing the current evidence, our work underscores the clinical relevance of inflammatory phenotyping and its potential to inform personalized treatment strategies. Furthermore, this review outlines directions for future research, emphasizing the importance of validating leukocyte-based biomarkers and developing targeted therapies that address specific inflammatory pathways in COPD.

## 7. Conclusions

COPD is a complex condition characterized by chronic inflammation, tissue damage, and airflow limitation. Understanding the diverse roles of inflammatory cell subtypes has significant clinical implications for its management. Neutrophils play a primary role in airway inflammation and tissue damage and represent potential therapeutic targets; strategies that specifically inhibit neutrophilic activation may reduce exacerbations and improve lung function. Patients with eosinophilic COPD may benefit from corticosteroids and biological therapies that target interleukin pathways, resulting in improved control of symptoms. The role of lymphocytes, particularly CD8^+^ T cells, suggests the need for immunomodulatory therapies that can restore the immune balance and mitigate chronic inflammation. Recognizing the involvement of monocytes and basophils in COPD pathogenesis can pave the way for innovative treatments that target these cells to alleviate inflammation and improve tissue repair mechanisms. A better understanding of the immunological underpinnings of COPD will enhance diagnostic accuracy through biomarkers and facilitate personalized treatment plans that address the specific inflammatory processes at play in each patient, ultimately leading to improved health outcomes. Future research should focus on targeted therapies that modulate specific inflammatory pathways and identify biomarkers to guide clinical decision making. By integrating immunological insights into clinical practice, we can develop more effective management strategies and enhance the quality of life of patients with COPD.

## Figures and Tables

**Table 1:** Clinical significance of leukocyte subtypes in COPD.

Leukocyte Subtypes	Clinical Significance
Neutrophils	Neutrophilic inflammation is commonly defined as a sputum neutrophil percentage ≥ 61% [7] or >64% [9]; Sputum neutrophil percentages >86.2% are associated with a higher GOLD stage, worse symptoms, and severe exacerbations [8]; 75% of patients had sputum neutrophil percentages >64%, and the mean percentages by GOLD group were A—66.5%, B—84.2%, C—72.1%, D—78.7% [9]; Neutrophilic exacerbation (neutrophils > 7000 cells/μL or >73%) is associated with higher mortality rates [11].
Lymphocytes	Increased CD8^+^ T cells and cytotoxic protein expression in COPD lung tissues [18]; Increased B cells and plasma cells in airways, correlating with disease severity [20]; Patients with CAT scores > 30 had the lowest CD4^+^ (21.8%) and highest CD8^+^ (53.1%) levels, with a lowest CD4^+^/CD8^+^ ratio of 0.43 [22]; Higher IL-17 levels (in serum or sputum) are associated with disease progression. Higher serum IL-17 in stable COPD vs. controls (SMD: 1.77) and in AECOPD vs. stable COPD (SMD: 1.78) [23]; Lymphocyte proportion ≤ 20% was linked to higher 3-year mortality [24]; Absolute counts < 0.8 × 10^9^/L were associated with higher mortality, longer hospital stays, and mechanical ventilation [25]
Neutrophil-to-lymphocyte ratio (NLR)	Higher NLR in patients with FEV_1_ < 50% (2.52) vs. ≥50% (2.06) and mMRC ≥ 2 (2.59) vs. <2 (2.06) [26]; NLR: non-survivors (3.0), survivors (2.3); higher NLR linked to increased all-cause mortality (hazard ratio = 1.16) [27]; NLR: 1.7 (healthy), 3.1 (stable COPD), 7.1 (exacerbation); cutoff 3.29 showed 80.8% sensitivity and 77.7% specificity for detecting exacerbation [28]; NLR ≥ 12.585 predicts hospitalization (sensitivity 89.5%, specificity 85.7); NLR ≥ 14.89 predicts mechanical ventilation (sensitivity 100%, specificity 96.2%) [29]
Monocytes	Higher serum IL-6 levels derived from CD14^+^ monocytes are associated with lower FEV1/FVC ratios and more exacerbations [35]; Monocyte proportion 7.4–10% linked to lowest exacerbation risk; <7.4% with <0.62 × 10^9^/L or >10% associated with higher risk [36]; Arginase activity increased during exacerbations (0.70 vs. 0.50 µmol urea/min in controls) and remained elevated for up to 3 months [37].
Monocyte-to-lymphocyte ratio (MLR)	MLR values were 0.23 (controls), 0.67 (stable COPD), and 0.91 (exacerbations); elevated MLR values were linked to higher exacerbation risk [38].
Eosinophils	A 3% eosinophil threshold identified eosinophilic inflammation in 22–46% of stable COPD patients [7,43]; Group C showed higher eosinophil levels (300 cells/µL) than group A (60 cells/µL) and group B (70 cells/µL) [9]; Higher eosinophil levels (≥170 cells/μL or >2%) are associated with steeper decline in FEV1, more exacerbations, disease progression, lung function decline, and disease severity [44,46]; Elevated eosinophils were linked to higher COPD exacerbation risk at 300 cells/μL (RR: 1.21), 400 cells/μL (RR: 1.79), 2% (RR: 1.26), and 4% (RR: 1.44) [47]; High eosinophil counts were associated with moderate-to-severe exacerbations at 300 cells/μL (RR: 1.30) and 2% (RR: 1.33) [47].
Basophils	An increased basophil count is observed in very severe COPD (stage IV) [56]; A higher basophil count is associated with poorer lung function and eosinophilic inflammation [57].

Abbreviations: COPD, chronic obstructive pulmonary disease; CAT, COPD assessment test; FEV1, forced expiratory volume in 1 s; FVC, forced vital capacity; mMRC, Modified Medical Research Council Dyspnea Scale; IL-6, interleukin-6; IL-17, interleukin-17; GOLD, Global Initiative for Chronic Obstructive Lung Disease; MLR, monocyte-to-lymphocyte ratio; NLR, neutrophil-to-lymphocyte ratio; RR, relative risk.

## Data Availability

No new data were created or analyzed in this study.
